
JOURNAL INFORMATION
==============================
NLM Title Abbreviation: Wirtschaftsdienst
Journal ID: Wirtschaftsdienst
NLM Journal ID: 101086612

Wirtschaftsdienst (Hamburg, Germany : 1949)

ISSN: 0043-6275

ARTICLE INFORMATION
==============================
PMCID: PMC8383024
PMID: 34456390
DOI: 10.1007/s10273-021-2989-z
Article ID: 2989
Article version: 1
Subjects: Ökonomische Trends

Konjunkturschlaglicht: Rohstoffpreise: Superzyklus oder Aufschwung?

Economic headline: Commodity prices: Supercycle or upswing?

Wellenreuther Claudia wellenreuther@hwwi.org

grid.435054.3 0000 0001 2227 8589 HWWI, Oberhafenstraße 1, 20097 Hamburg, Deutschland
Electronic publication date: 2021 Aug 24
Print publication date: 2021
Volume: 101
Issue: 8
First page: 663
Last page: 664
Copyright: © Der/die Autor:in(nen) 2021
License: Open Access: Dieser Artikel wird unter der Creative Commons Namensnennung 4.0 International Lizenz veröffentlicht (https://creativecommons.org/licenses/by/4.0/deed.de).
Open Access wird durch die ZBW — Leibniz-Informationszentrum Wirtschaft gefördert.
License URL: https://creativecommons.org/licenses/by/4.0/

issue-copyright-statement: © The Author(s) 2021

==============================

Literatur

Cuddington J Jerrett D Super Cycles In Real Metals Prices? IMF Staff Paper 2008 55 4 541 565 10.1057/imfsp.2008.19
Delle Chiaie, S., L. Ferrara und D. Giannone (2017), Common factors of commodity prices, ECB Working Paper, 2112.
Erten, B. und J. A. Ocampo (2012), Super-Cycles of Commodity Prices Since the Mid-Nineteenth Century, DESA Working Paper, 110, Februar, ST/ESA/DWP/110.
Heap, A. (2005), China — The Engine of a Commodities Super Cycle, Citigroup Smith Barney.
Schmidt, T., F. Kirsch und M. Dirks (2021), Kurzfristige Perspektiven der Rohstoffpreisentwicklung, Gutachten im Auftrag des Ministeriums für Wirtschaft, Innovation, Digitalisierung und Energie des Landes Nordrhein-Westfalens, RWI — Leibniz-Institut für Wirtschaftsforschung, Juli.
